# Supplementary material for: APOE genotype and sex drive microbiome divergence after microbiome standardization in APOE-humanized mice
Source: mSphere. 2025 Oct 20;10(11):e00429-25. doi: 10.1128/msphere.00429-25 (PMC12645986; doi:10.1128/msphere.00429-25)
Supplement: Supplemental material — Supplemental figures and tables. [file msphere.00429-25-s0001.docx]

**Supplementary Information**

*APOE* genotype and sex drive microbiome divergence after microbiome standardization in *APOE*-humanized mice

Michelle Aries Marchington^1,2^, Hope Gasvoda^1^, Makayla Michelotti^1^, Fernando Rodriguez-Caro^3^, Ashley Gooman^1,4^, Anna Perez^5^, Tiffany Hensley-McBain*^1,2^

1. McLaughlin Research Institute, Weissman Hood Institute at Touro University, Great Falls, MT USA
2. Touro College of Osteopathic Medicine Montana, Great Falls, MT USA
3. University of Montana, Missoula, MT USA
4. University of Providence, Great Falls, MT USA
5. Touro College of Osteopathic Medicine Harlem, NY, USA

*** Correspondence:**

Tiffany Hensley-McBain, PhD
[thmcbain@mclaughlinresearch.org](mailto:thmcbain@mclaughlinresearch.org)

**Table of Contents**

**Cages/sex/genotype Table**

Sup. Table. 1. Displays the number of cages per sex per genotype for experimental mice…………………...………...………………………………….…….3

**Abundance and Principal Component Analysis (PCA)**

Sup. Fig. 1. A. Bacterial Abundance plot of B6 mothers before and after breeding and APOE 2, 3, and 4 fathers B. PCA plot of B6 mothers before and after breeding, offspring, and APOE 2, 3, and 4 fathers…….………..…………….…….4

**Abundance Plots**

Sup. Fig. 2. At wean and 6-month-old females and males with grandparents A. Homozygous B. Heterozygous…….………...………………………………….…….5

**Principal Component Analysis (PCA) for 6-month-old APOE 2,3, and 4 mice**

Sup. Fig. 3. A. 6-month-old heterozygous females with APOE fathers 2,3,4 and C57BL/6J mothers (parents) B. 6-month-old heterozygous males with parents C. At wean homozygous females with APOE grandfathers 2,3,4 and C57BL/6J grandmothers (grandparents) D. At wean homozygous males with grandparents E. 6-month-old homozygous females with grandparents F. 6-month-old homozygous males with grandparents..………………..………………………….....6

**Linear discriminant analysis effect size (LEfSe) of 6-month-old heterozygous mice**

Sup. Fig. 4. LEfSe showing bacteria indicative of APOE genotype. A. females B. males.……………………………………………………………………………..….…..7

**Shannon Diversity**

Sup. Fig. 5. A. 6-month-old heterozygous females and males and B. 6-month-old homozygous females and males ..…………………………………………….……...8

**Dot Plot** of statistically significant bacteria from the top 100 bacterial species with a 2-way ANOVA followed by Tukey’s multiple comparisons test with multiplicity adjusted p-values

Sup. Fig. 6. 6-month-old homozygous A. females and B. males..……..................9

**Cladogram of bacteria indicative of APOE genotype generated by LEfSe**

Sup. Fig. 7. 6-month-old homozygous A. females B. males.………….……….…10

Sup. Fig. 8. 6-month-old heterozygous A. females B. males.………….………...11

**Dot Plot** with a 2-way ANOVA followed by Tukey’s multiple comparisons test with multiplicity adjusted p-values

Sup. Fig. 9. 6-month-old homozygous A. high abundance families and B Low abundance families……………………………………………………………….…...12

**Coda4microbiome Analysis for 6-month-old APOE 2,3, and 4 mice**

Sup. Fig. 10. Microbial signatures differentiating *APOE* genotypes A. APOE3 vs APOE2 females. B. APOE4 vs APOE2 females. C. APOE4 vs APOE3 females. D. APOE3 vs APOE2 males. E. APOE4 vs APOE2 males……………………..…13

**LEfSe Tables**

Sup. Table 2. Indicative bacterial list from the LEfSe analysis for homozygous 6-month-old female mice..………………………..………………………...............14-15

Sup. Table 3. Indicative bacterial list from the LEfSe analysis for homozygous 6-month-old male mice………………………..….………………..…….................16-17

| **Genotype** | **Sex** | **Number of Cages** |
| --- | --- | --- |
| Hmz APOE4 | Female | 3 |
|  | Male | 3 |
| Hmz APOE3 | Female | 3 |
|  | Male | 3 |
| Hmz APOE2 | Female | 3 |
|  | Male | 3 |
| Het APOE4 | Female | 3 |
|  | Male | 2 |
| Het APOE3 | Female | 2 |
|  | Male | 2 |
| Het APOE2 | Female | 2 |
|  | Male | 2 |

**Supplementary Table 1. Number of cages/sex/genotype for experimental mice**


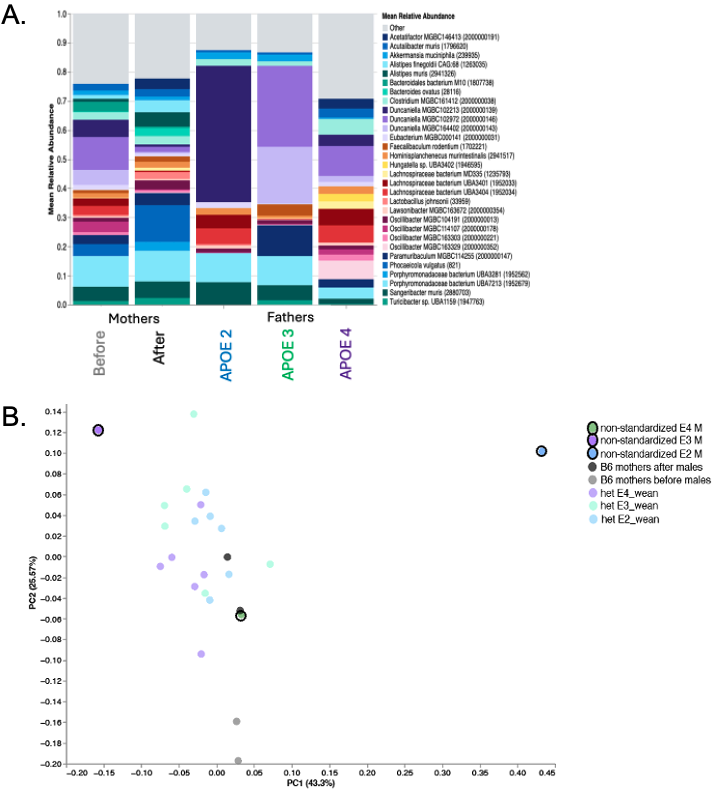


**Supplementary Figure 1. Comparison of B6 mothers before breeding and after the last breeding**. A. Top 30 bacterial species taxa plots for B6 mothers before and after breeding, and APOE 2, 3, and 4 fathers. B. Principal Component Analysis (PCA) for B6 mothers before and after breeding, APOE 2, 3, and 4 fathers, and heterozygous pups at wean. n=1/genotype for fathers, n=2 for the mothers, n=3/sex/genotype for the pups.


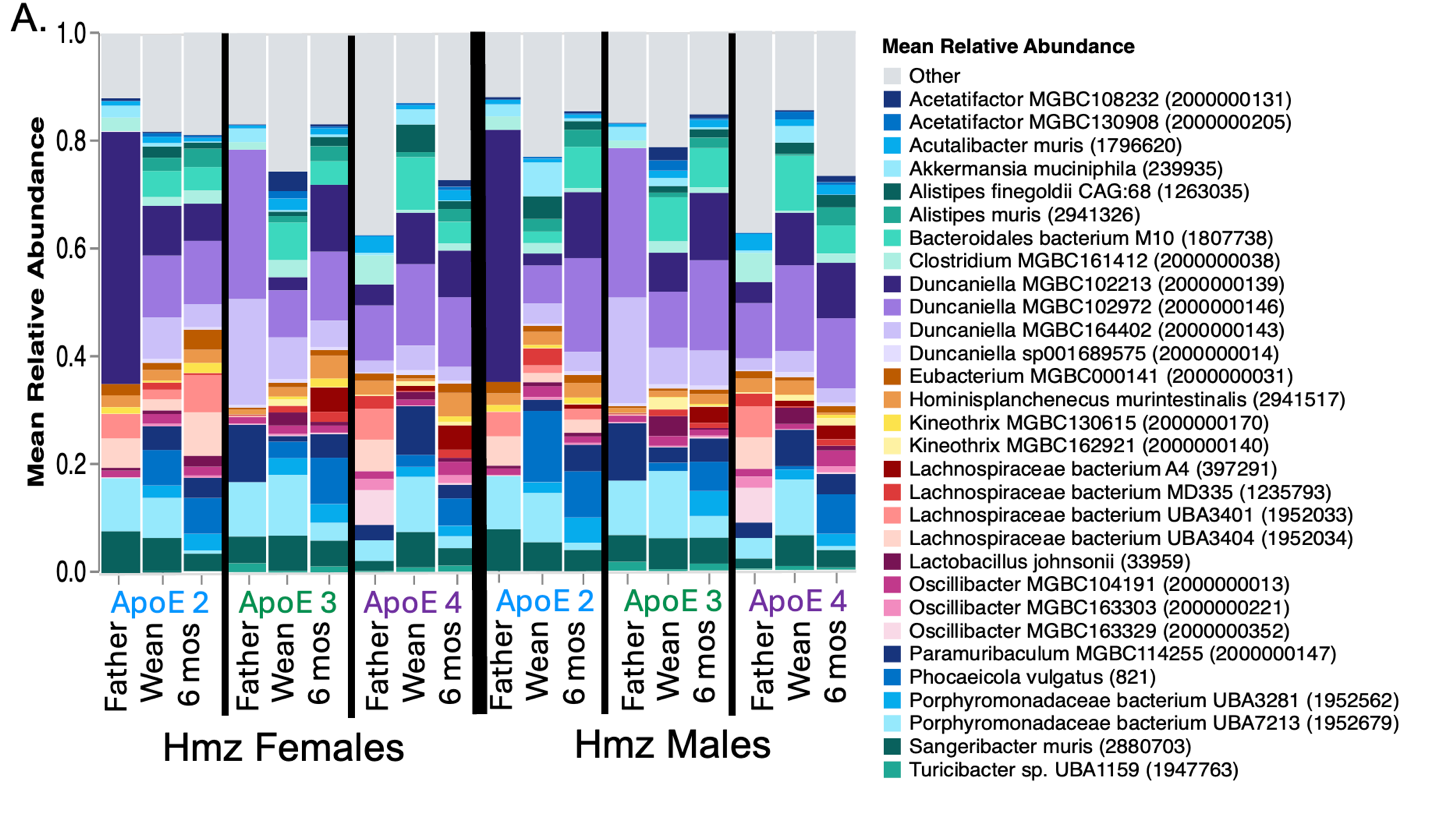

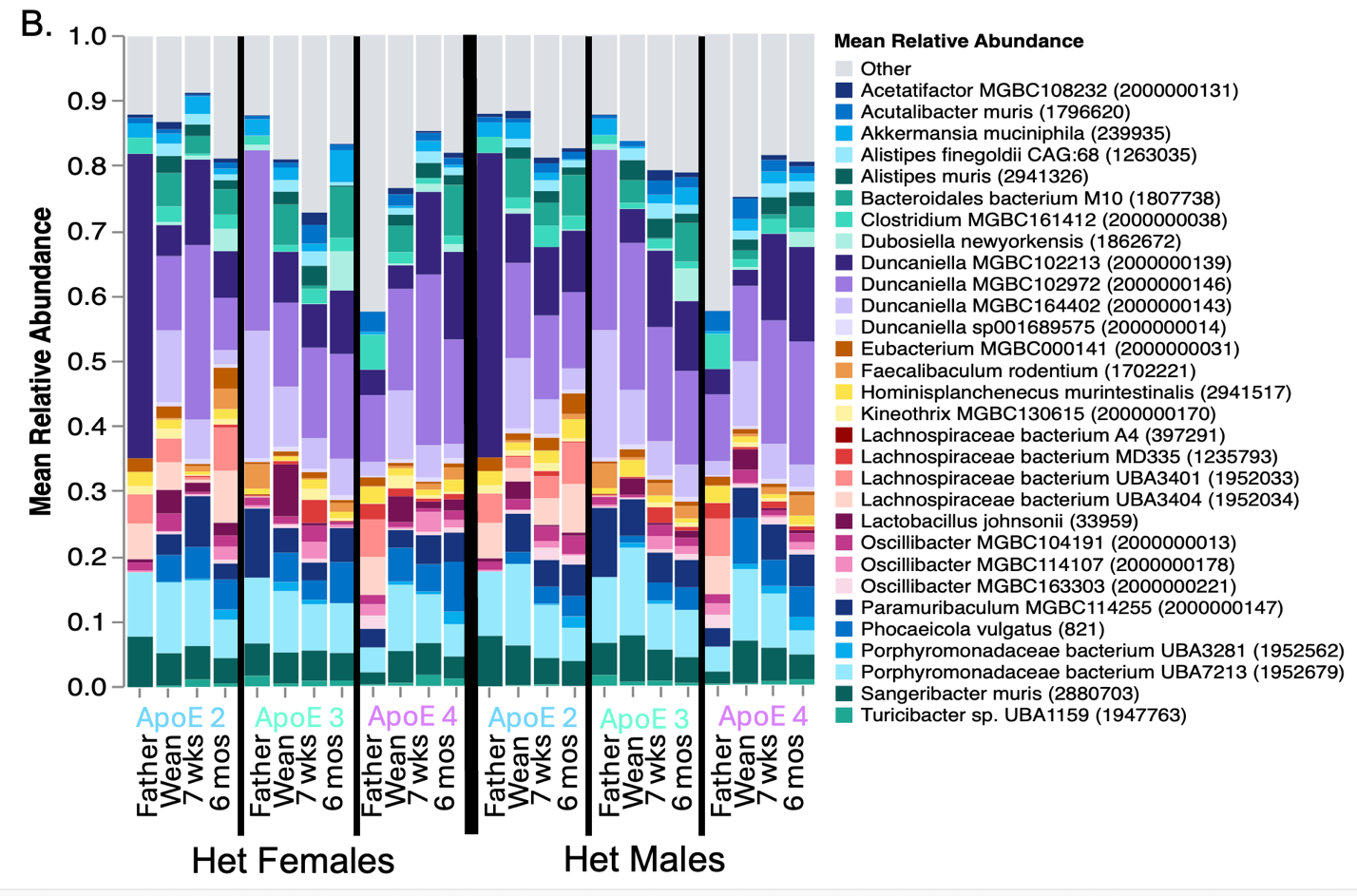


**Supplementary Figure 2. Top 30 bacterial species taxa plots for microbiome data on humanized APOE 2, 3, and 4 mice.** A. Homozygous female and male with their grandfathers (males from JAX) at wean and 6-months-old. B. Heterozygous female and male with their fathers (males from JAX) at wean, 7-weeks-old, and 6-months-old. n=3/sex/genotype for hets, n=5/sex/genotype for hmz, n=1/genotype for fathers.


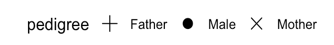

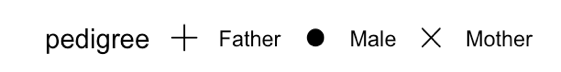

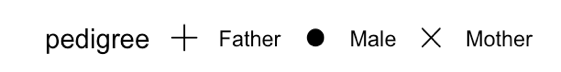

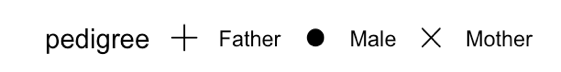

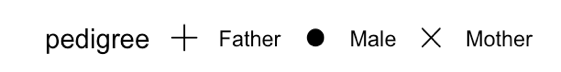

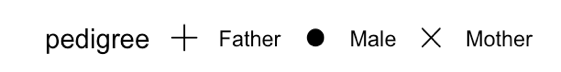

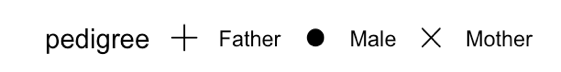

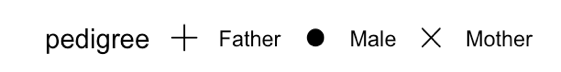

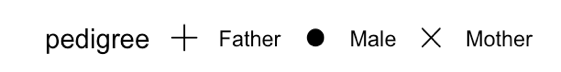

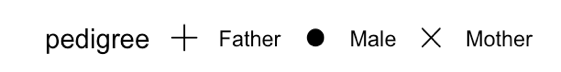

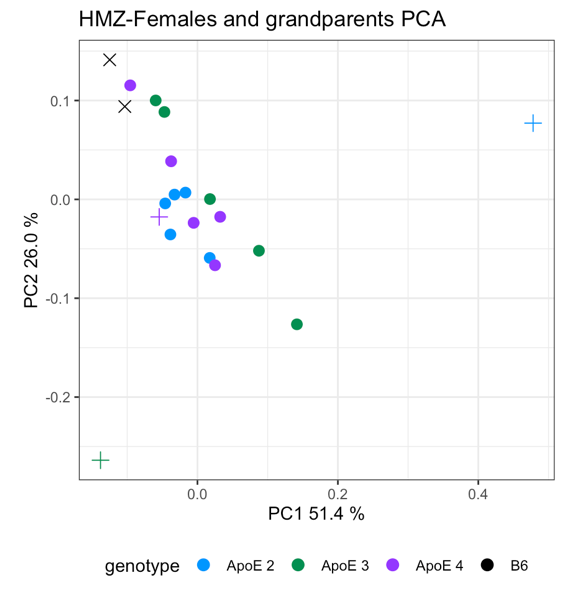

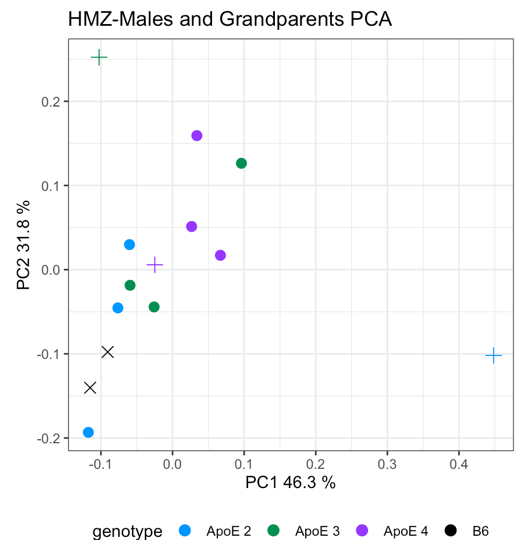

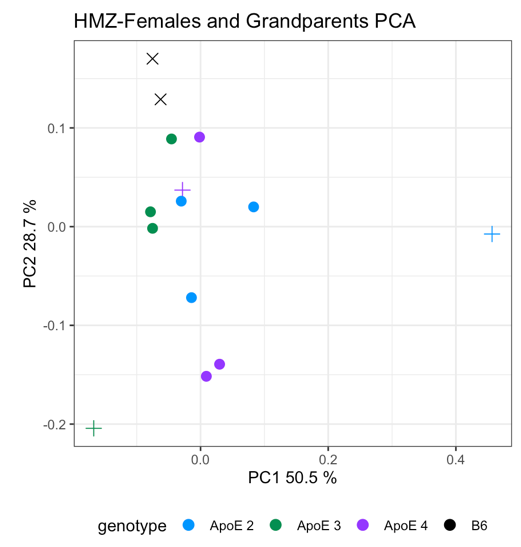

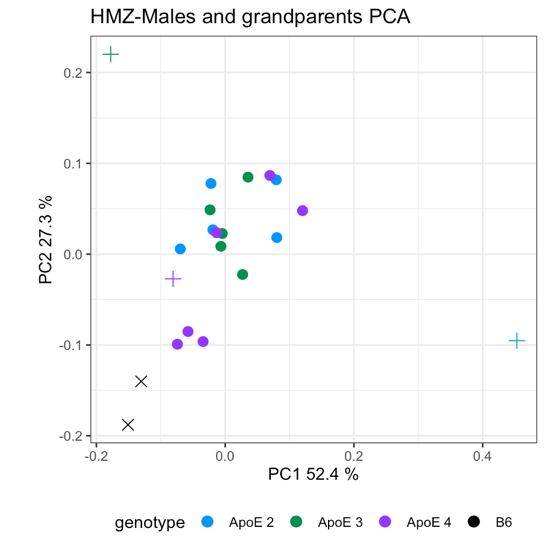


Females

A.

B.

C.

D.

E.

F.


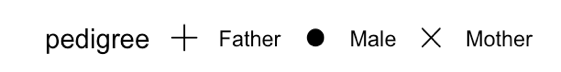

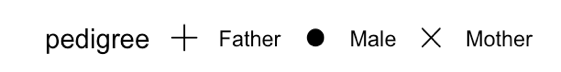


Males


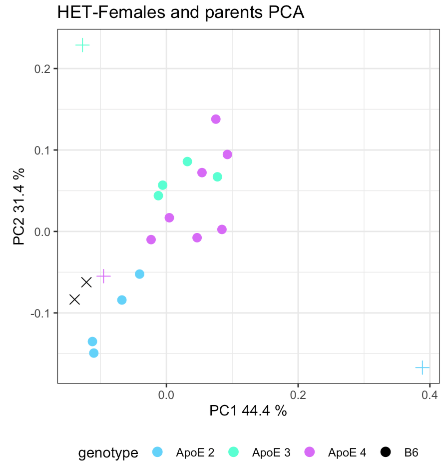

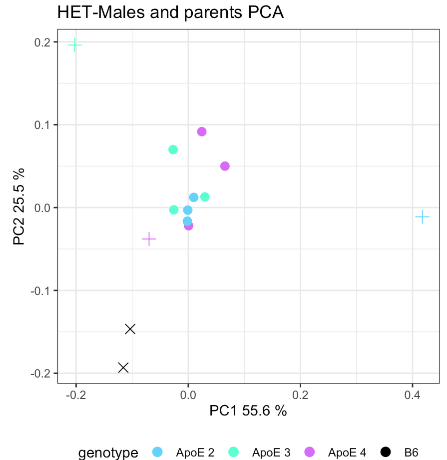


**Supplementary Figure 3. Principal Component Analysis (PCA) APOE 2,3, and 4 mice.** A. 6-month-old heterozygous females with APOE fathers 2,3,4 and C57BL/6J mothers (parents) B. 6-month-old heterozygous males with parents C. At wean homozygous females with APOE grandfathers 2,3,4 and C57BL/6J grandmothers (grandparents) D. At wean homozygous males with grandparents E. 6-month-old homozygous females with grandparents F. 6-month-old homozygous males with grandparent. n=3/sex/genotype for hets, n=5/sex/genotype for hmz, n=1/genotype for fathers, n=2 for mothers.

B.

A.

**Supplementary Figure 4. Linear discriminant analysis effect size (LEfSe) for 6-month-old heterozygous humanized APOE 2, 3, and 4 mice, demonstrating bacteria indicative of APOE 2, 3, or 4 genotype.** A. Females B. Males. Only bacteria with an LDA score > 2 and p < 0.05 are shown. n=3/sex/genotype


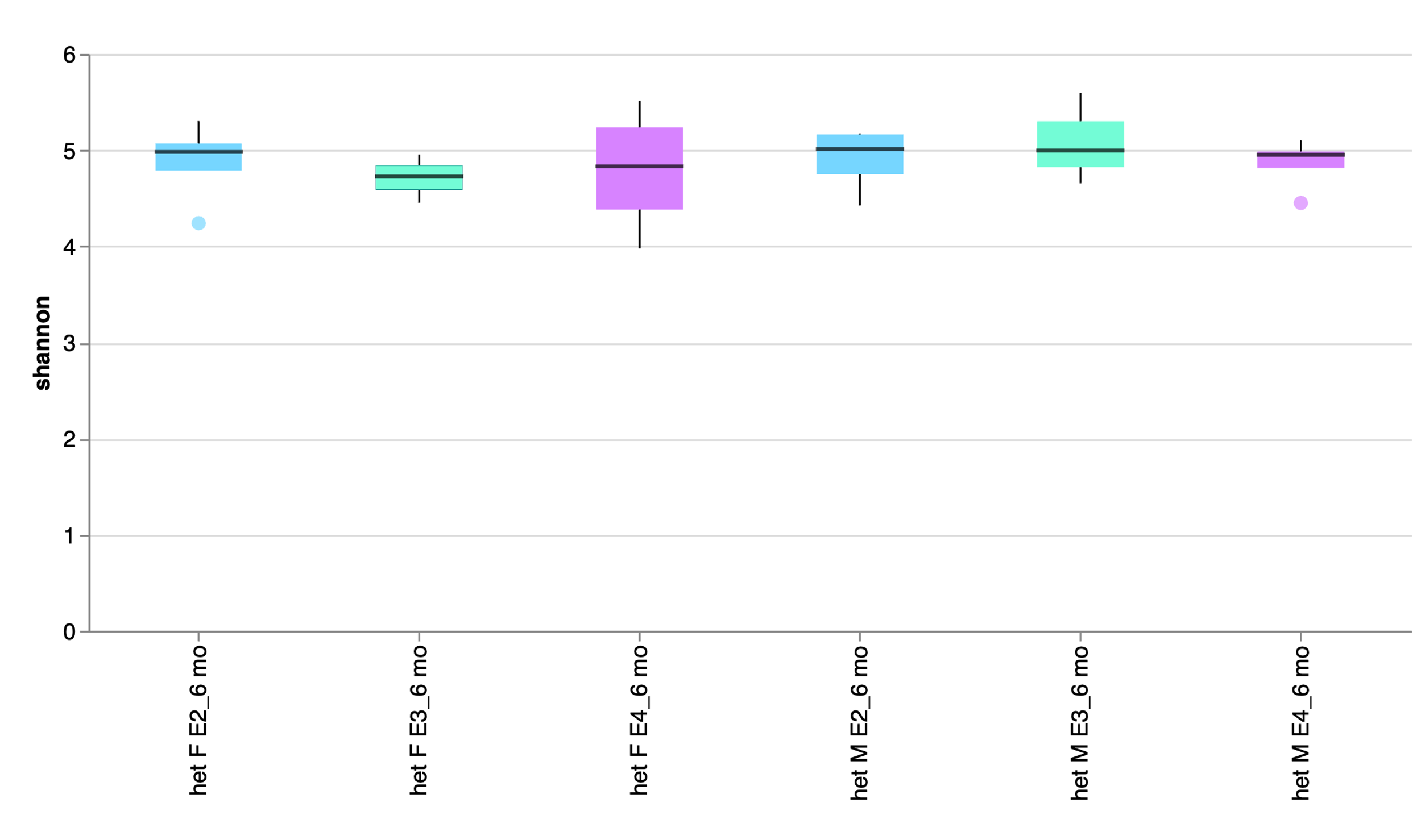


A.v

Het APOE2 Het APOE3 Het APOE4

Het APOE2 Het APOE3 Het APOE4

Female

Male


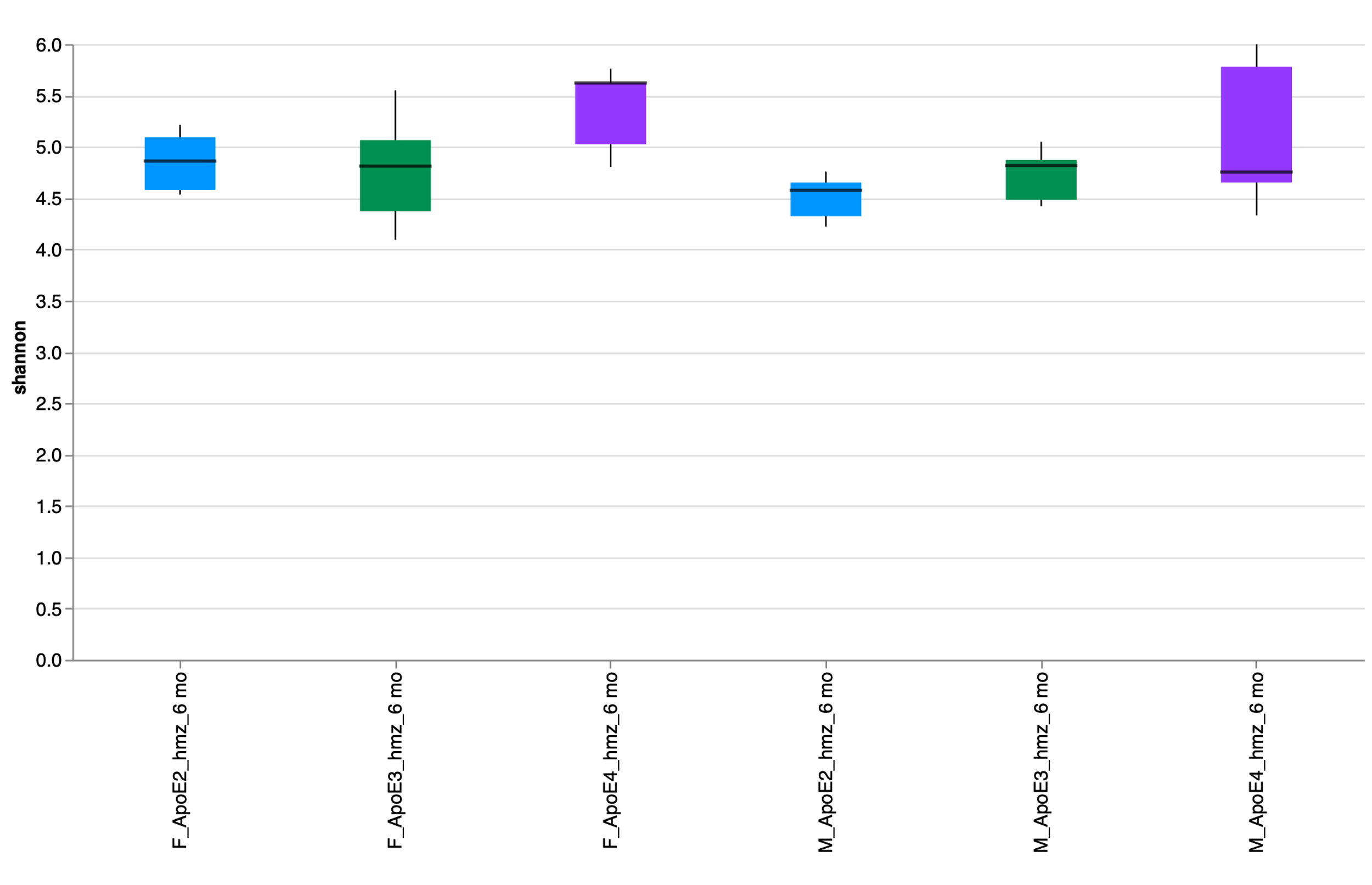


B.v

Male

Female

APOE2 APOE3 APOE4

APOE2 APOE3 APOE4

**Supplementary Figure 5. Shannon Diversity Plot of APOE 2,3, and 4 mice.** A. 6-month-old heterozygous females and males. n=5/sex/genotype B. 6-month-old homozygous females and males. n=3/sex/genotype


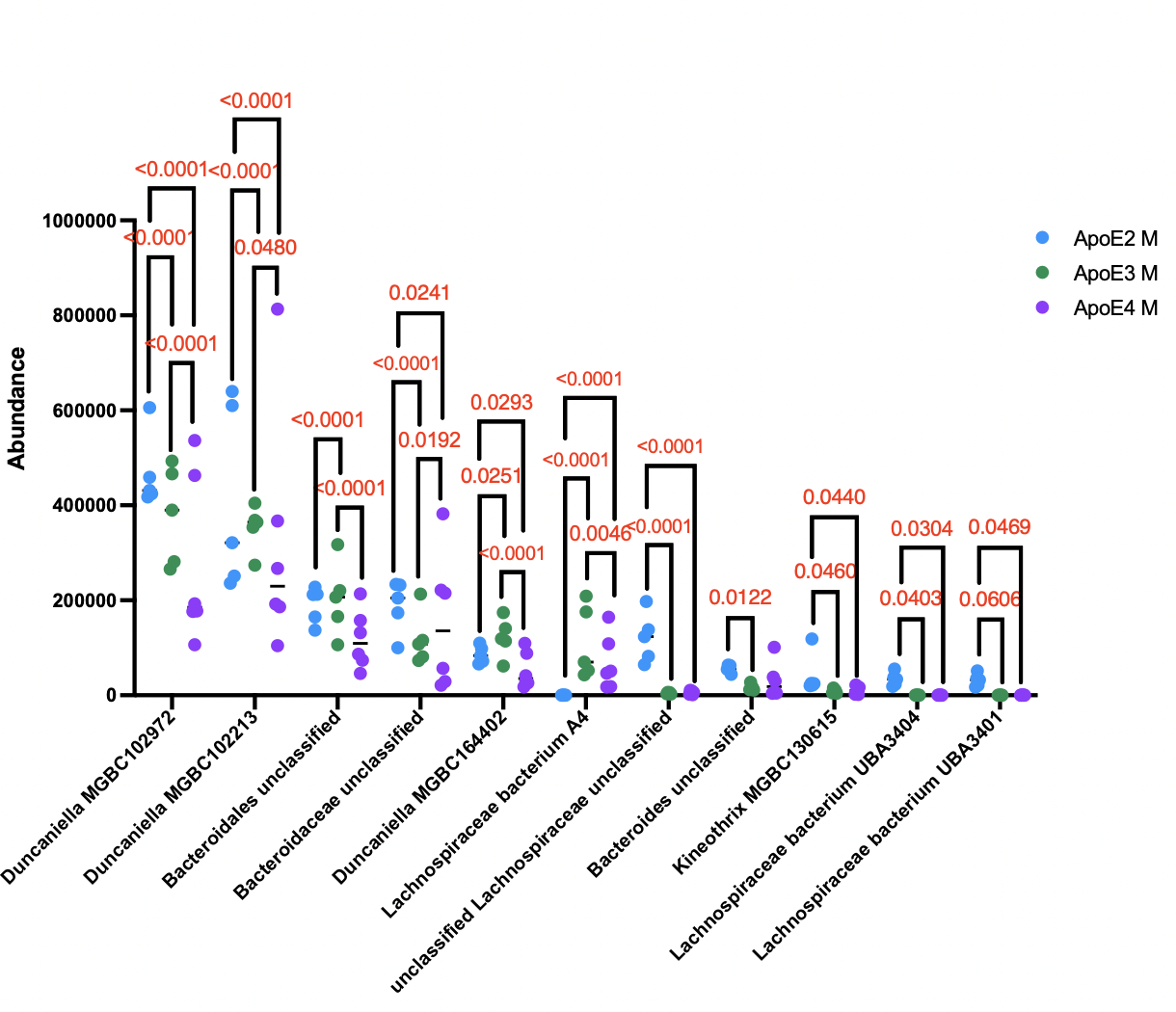

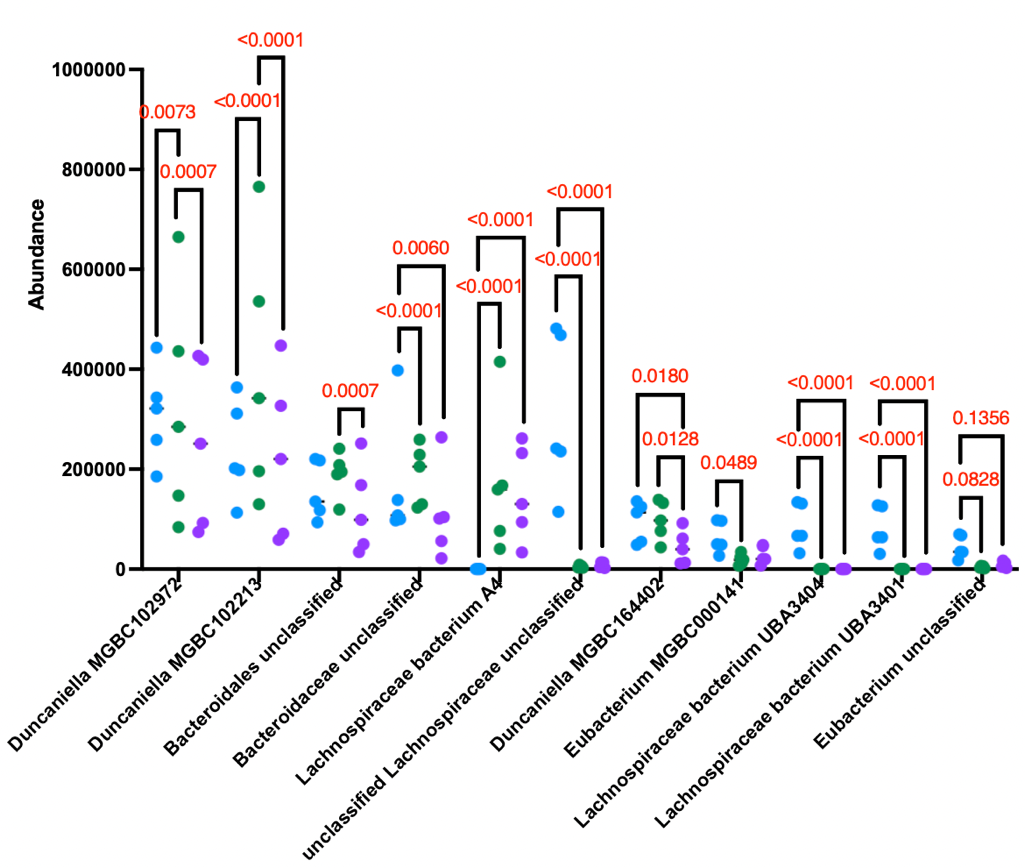


A.v

B.v

**Supplementary Figure 6. Divergent bacterial species among genotypes.** A. Homozygous Females B. Homozygous Males. Bacterial species with statistical significance assessed by 2-way ANOVA followed by Tukey’s multiple comparisons test with multiplicity adjusted p values. Only species with p value < 0.05 are shown. Species representing greater than 0.005% of total abundance were included in analyses. Species represented in the bottom 125 species are not shown. n=5/sex/genotype

**Supplementary Figure 7. Cladogram of shallow shotgun microbiome data for 6-month-old homozygous humanized APOE 2, 3, and 4 mice, demonstrating the relationship between bacteria that are indicative of APOE 2, 3 or 4 genotype.** A. Females B. Males. Only bacteria with an LDA score > 2 and p < 0.05 are shown. n=5/sex/genotype

APOE2M

APOE3M

APOE4M

APOE2 F

APOE3 F

APOE4 F

A.


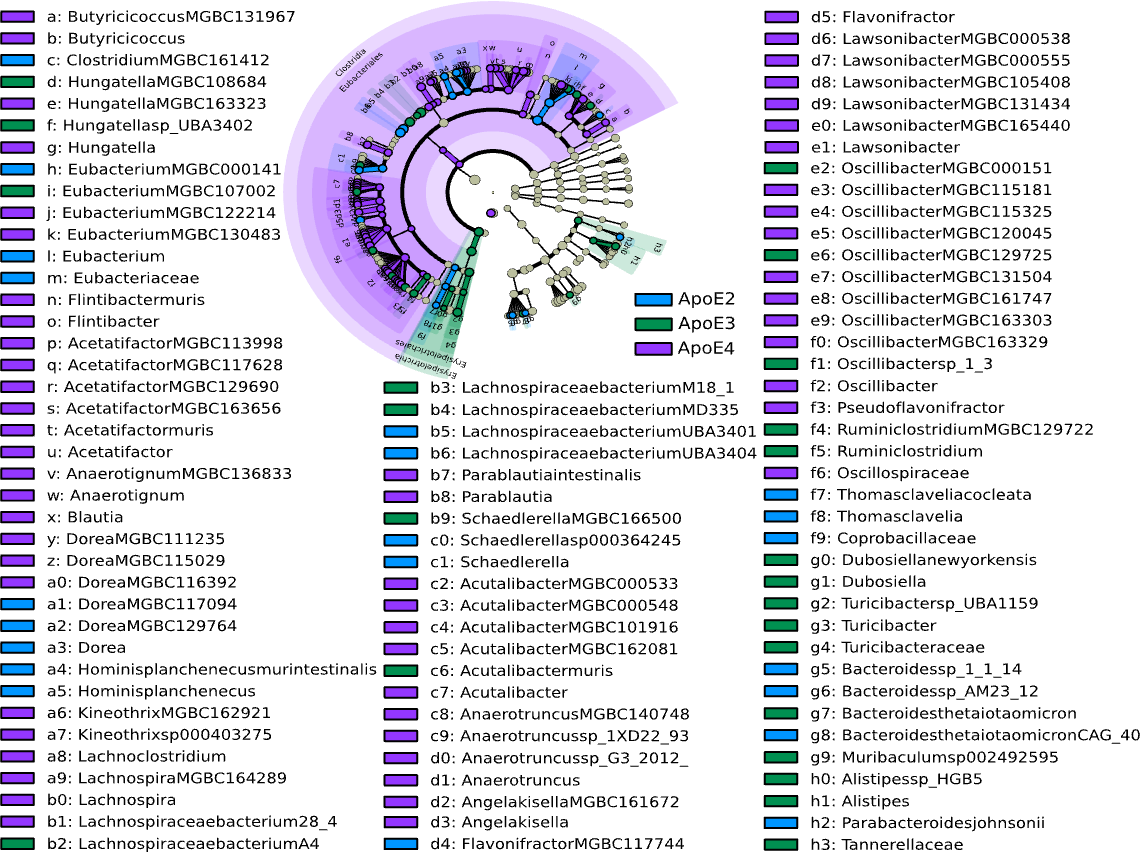

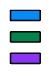


B.


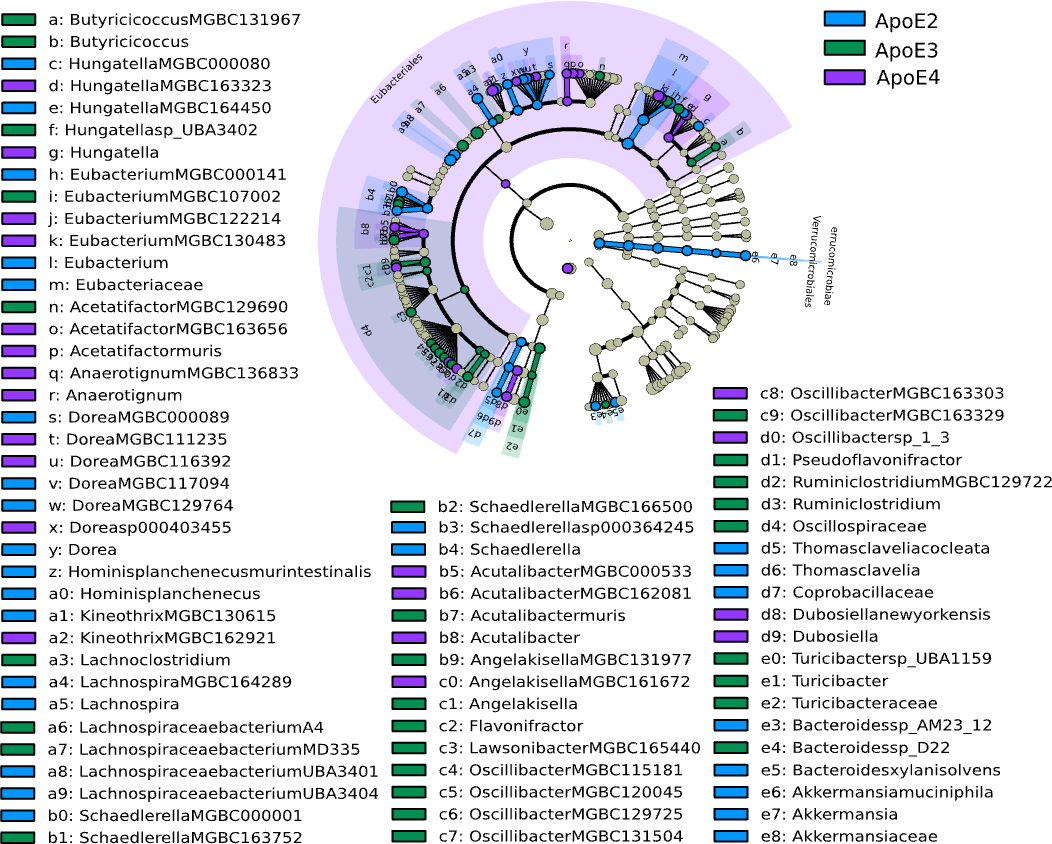

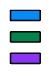


APOE2 M

APOE3 M

APOE4 M

APOE2 M

APOE3 M

APOE4 M

B.

APOE2 F

APOE3 F

APOE4 F

A.

**Supplementary Figure 8. Cladogram of shallow shotgun microbiome data for 6-month-old heterozygous humanized APOE 2, 3, and 4 mice, demonstrating the relationship between bacteria that are indicative of APOE 2, 3 or 4 genotype.** A. Females B. Males. Only bacteria with an LDA score > 2 and p < 0.05 are shown. n=3/sex/genotype


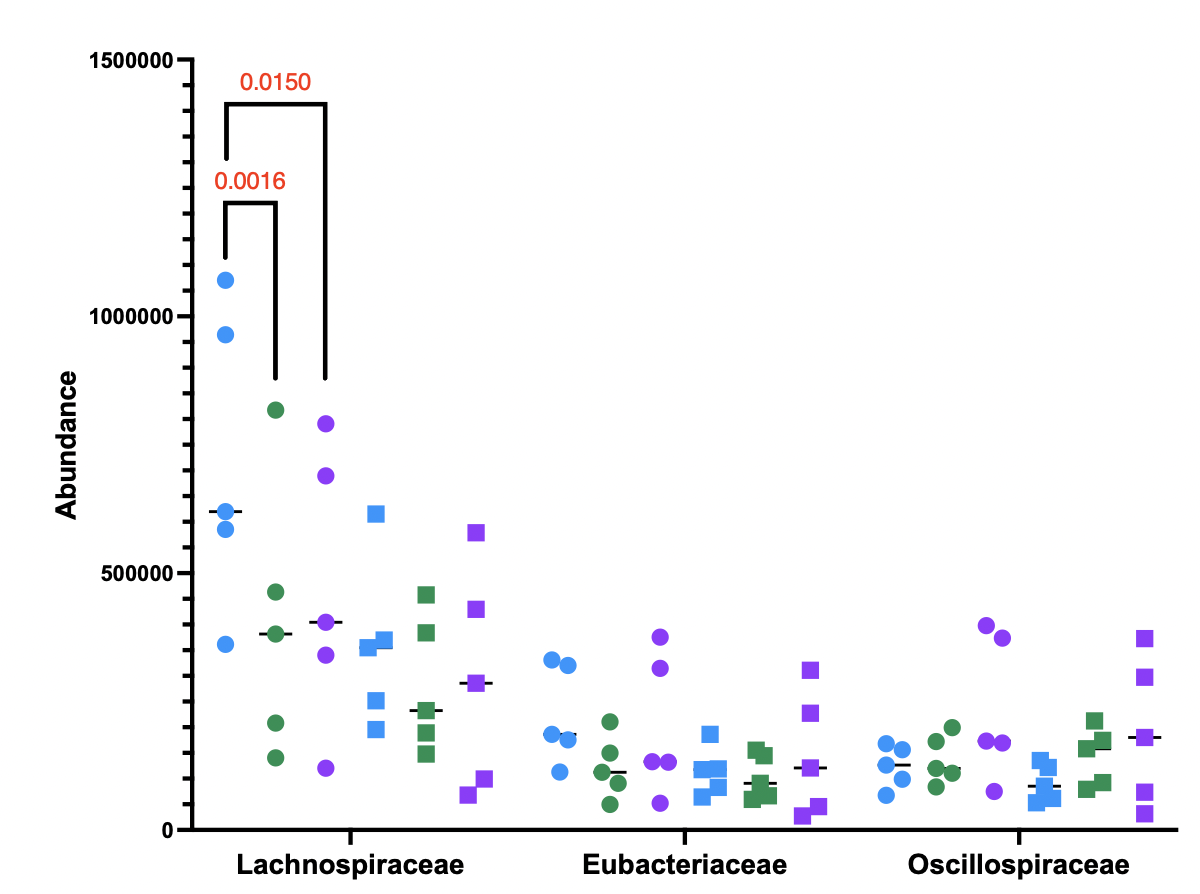

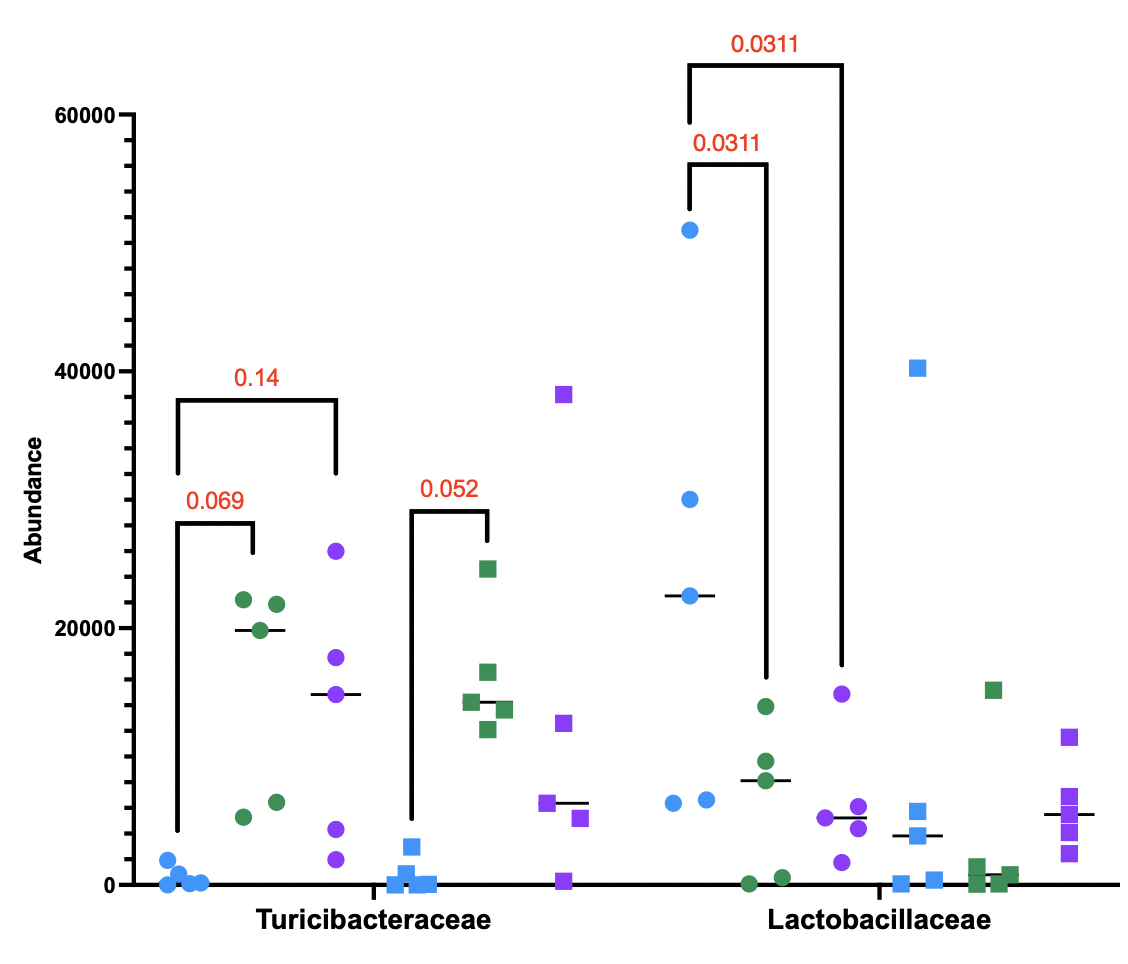


B.v


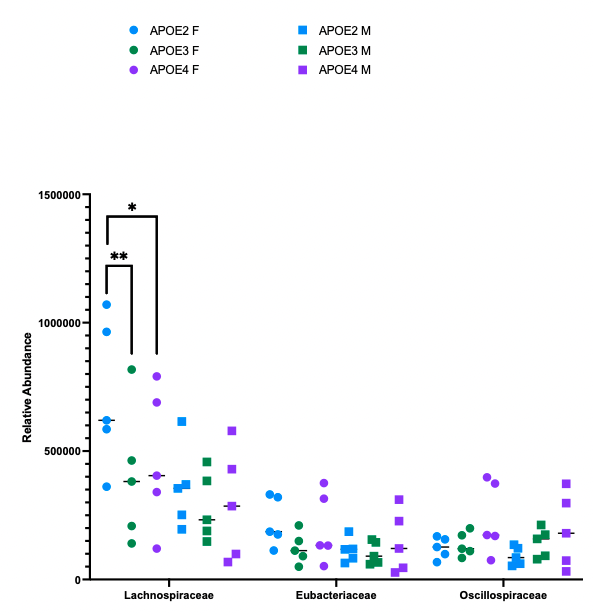

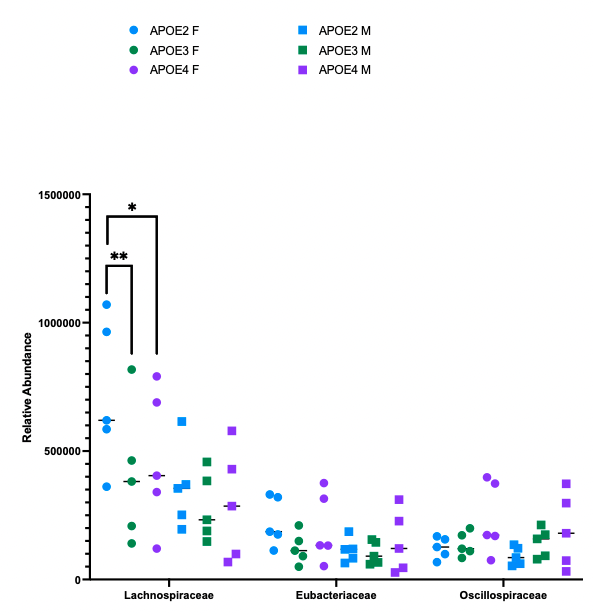

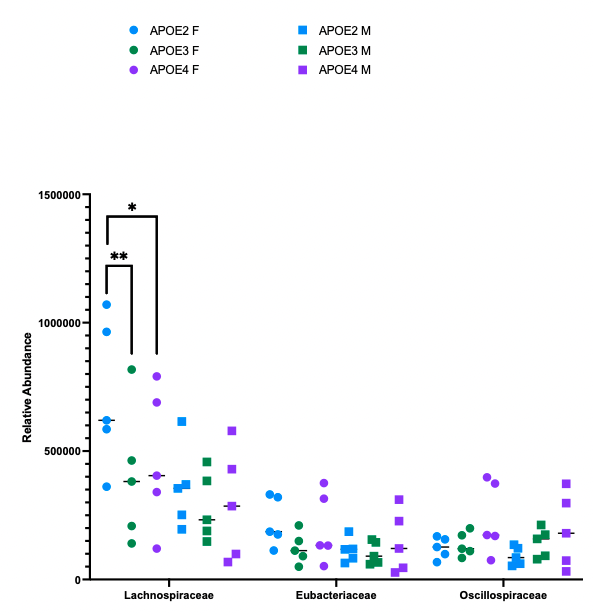

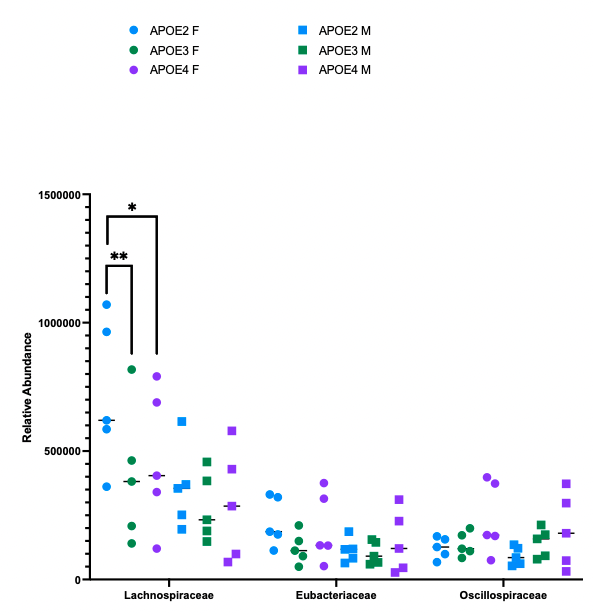


A.v

**Supplementary Figure 9. Divergent bacterial families among genotypes in homozygous females and males.** A. High abundance bacteria families. B. Low abundance bacteria families. Bacterial families with statistical significance assessed by 2-way ANOVA followed by Tukey’s multiple comparisons test with multiplicity adjusted p values. n=5/sex/genotype


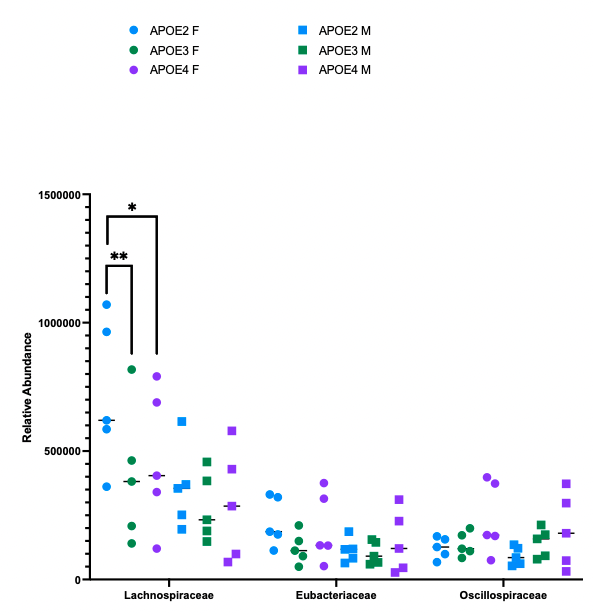


Females

D.

E.

Males


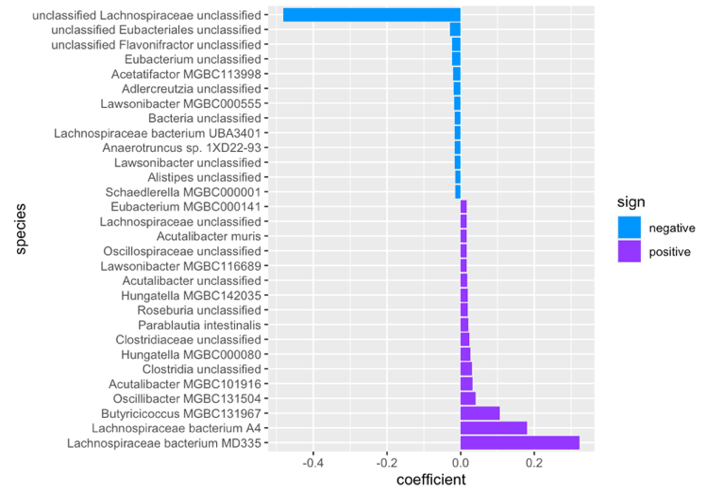

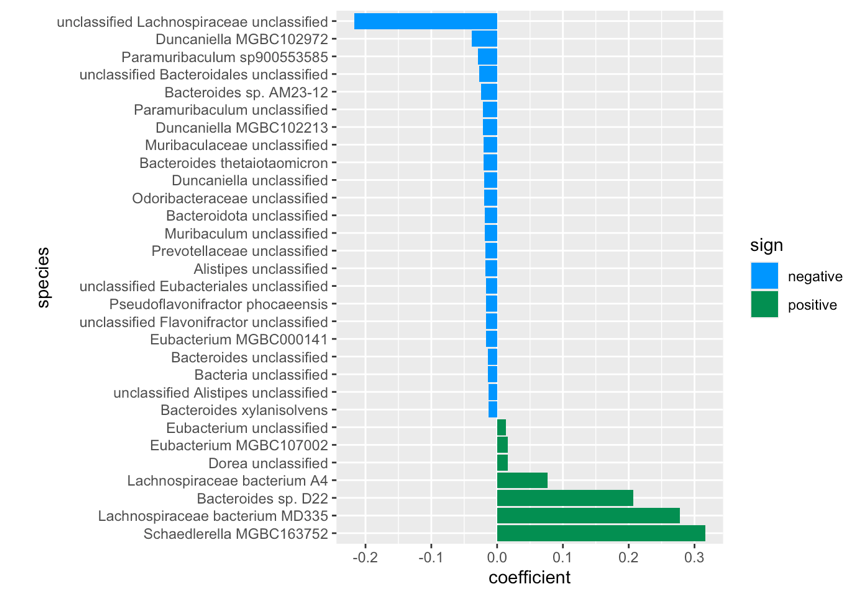

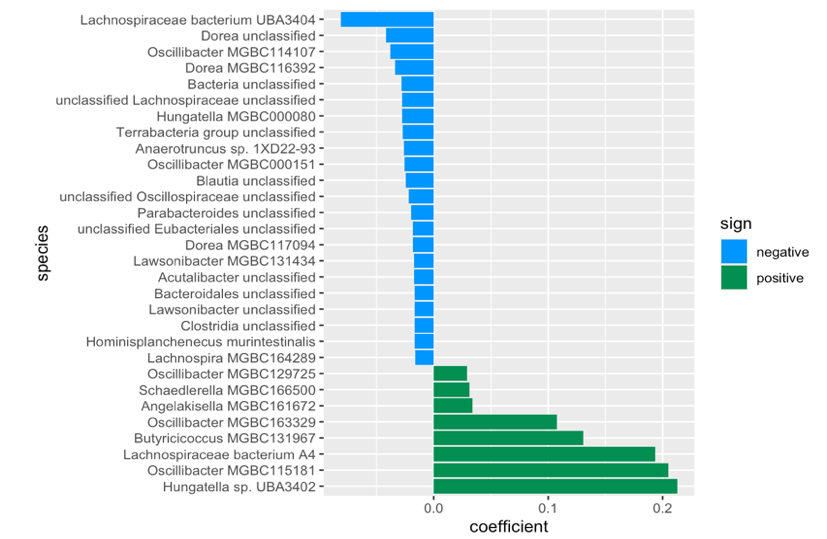

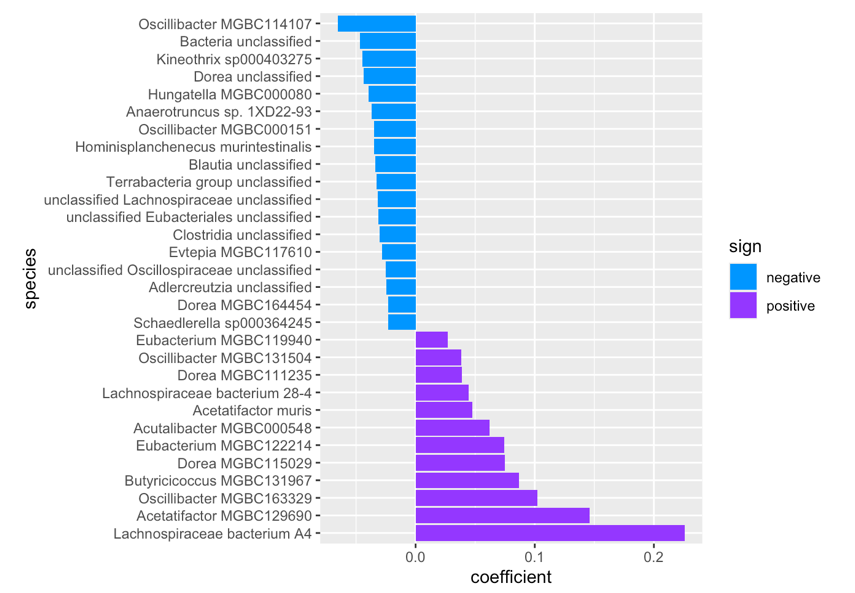

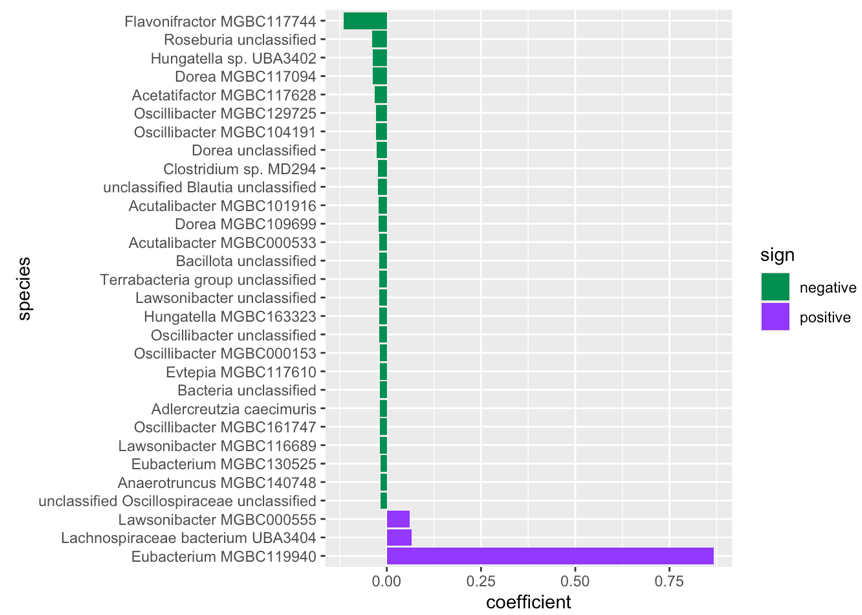


A.

B.

C.

**Supplementary Figure 10. Microbial signatures differentiating *APOE* genotypes of 6-month-old homozygous *APOE* 2,3 and 4 mice.** Coda4microbiome analysis showing bacteria comprising discriminatory microbial signatures between APOE genotypes. A. APOE3 vs APOE2 females. B. APOE4 vs APOE2 females. C. APOE4 vs APOE3 females. D. APOE3 vs APOE2 males. E. APOE4 vs APOE2 males. n=5/sex/genotype

| **Supplementary Table 2. Indicative bacterial list from the LEfSe analysis for homozygous 6-month-old** **female mice.** Only bacteria with an LDA score>2 and p<0.05 are shown. n=5/genotype | | | |
| --- | --- | --- | --- |
| **Sex and Genotype** | **Family** | **Genus** | **Species** |
| **APOE 4 Females** | *Clostridiaceae* | *Hungatella* | *Hungatella MGBC163323* |
|  |  |  | *-* |
|  | *Eubacteriaceae* | *Eubacterium* | *Eubacterium MGBC130483* |
|  |  |  | *Eubacterium MGBC122214* |
|  | *Lachnospiraceae* | *Acetatifactor* | *Acetatifactor MGBC117628* |
|  |  |  | *Acetatifactor muris* |
|  |  |  | *Acetatifactor MGBC113998* |
|  |  |  | *Acetatifactor MGBC129690* |
|  |  |  | *Acetatifactor MGBC163656* |
|  |  |  | *-* |
|  |  | *Anaerotignum* | *Anaerotignum MGBC136833* |
|  |  |  | *-* |
|  |  | *Blautia* | *-* |
|  |  | *Parablautia* | *Parablautia intestinalis* |
|  |  |  | *-* |
|  |  | *Dorea* | *Dorea MGBC116392* |
|  |  |  | *Dorea MGBC111235* |
|  |  |  | *Dorea MGBC115029* |
|  |  | *Kineothrix* | *Kineothrix sp 000403275* |
|  |  |  | *Kineothrix MGBC162921* |
|  |  | *Lachnospira* | *Lachnospira MGBC164289* |
|  |  |  | *-* |
|  |  | *unclassified genus* | *Lachnospiraceae bacterium 28-4* |
|  | *Oscillospiraceae* | *Acutalibacter* | *Acutalibacter MGBC000533* |
|  |  |  | *Acutalibacter MGBC000548* |
|  |  |  | *Acutalibacter MGBC101916* |
|  |  |  | *-* |
|  |  |  | *Acutalibacter MGBC162081* |
|  |  | *Angelakisella* | *Angelakisella MGBC161672* |
|  |  |  | *-* |
|  |  | *Anaerotruncus* | *Anaerotruncus sp G3-2012* |
|  |  |  | *Anaerotruncus sp 1XD22-93* |
|  |  |  | *Anaerotruncus MGBC140748* |
|  |  |  | *-* |
|  |  | *Butyricicoccus* | *Butyricicoccus MGBC131967* |
|  |  |  | *-* |
|  |  | *Flavonifractor* | *-* |
|  |  | *Flintibacter* | *Flintibacter muris* |
|  |  |  | *-* |
|  |  | *Lawsonibacter* | *Lawsonibacter MGBC165440* |
|  |  |  | *Lawsonibacter MGBC000538* |
|  |  |  | *Lawsonibacter MGBC105408* |
|  |  |  | *Lawsonibacter MGBC131434* |
|  |  |  | *Lawsonibacter MGBC000555* |
|  |  |  | *-* |
|  |  | *Pseudoflavonifractor* | *-* |
|  |  | *Oscillibacter* | *Oscillibacter MGBC163303* |
|  |  |  | *Oscillibacter MGBC115181* |
|  |  |  | *Oscillibacter MGBC161747* |
|  |  |  | *Oscillibacter MGBC115325* |
|  |  |  | *Oscillibacter MGBC131504* |
|  |  |  | *Oscillibacter MGBC163329* |
|  |  |  | *Oscillibacter MGBC120045* |
|  |  |  | *-* |
| ***APOE3* Females** | *Bacteroidaceae* | *Bacteroides* | *Bacteroides thetaiotaomicron* |
|  | *Clostridiaceae* | *Hungatella* | *Hungatella sp UBA3402* |
|  |  |  | *Hungatella MGBC108684* |
|  | *Erysipelotrichaceae* | *Dubosiella* | *Dubosiella newyorkensis* |
|  |  |  | *-* |
|  | *Eubacteriaceae* | *Eubacterium* | *Eubacterium MGBC107002* |
|  | *Lachnospiraceae* | *Schaedlerella* | *Schaedlerella MGBC166500* |
|  |  | *Unclassified genus* | *Lachnospiraceae bacterium MD335* |
|  |  |  | *Lachnospiraceae bacterium A4* |
|  |  |  | *Lachnospiraceae bacterium M18-1* |
|  | *Muribaculaceae* | *Muribaculum* | *Muribaculum sp 002492595* |
|  | *Oscillospiraceae* | *Acutalibacter* | *Acutalibacter muris* |
|  |  | *Oscillibacter* | *Oscillibacter sp 1-3* |
|  |  |  | *Oscillibacter MGBC129725* |
|  |  |  | *Oscillibacter MGBC000151* |
|  |  | *Ruminiclostridium* | *Ruminiclostridium MGBC129722* |
|  |  |  | *-* |
|  | *Rikenellaceae* | *Alistipes* | *Alistipes sp HGB5* |
|  |  |  | *-* |
|  | *Tannerellaceae* | *-* | *-* |
|  | *Turicibacteraceae* | *Turicibacter* | *Turicibacter sp UBA1159* |
|  |  |  | *-* |
|  |  | *-* |  |
| **APOE2 Females** | *Bacteroidaceae* | *Bacteroides* | *Bacteroides sp AM23-12* |
|  |  |  | *Bacteroides sp 1-1-14* |
|  |  |  | *Bacteroides thetaiotaomicron CAG-40* |
|  | *Clostridiaceae* | *Clostridium* | *Clostridium MGBC161412* |
|  | *Coprobacillaceae* | *Thomasclavelia* | *Thomasclavelia cocleata* |
|  |  |  | *-* |
|  |  | *-* | *-* |
|  | *Eubacteriaceae* | *Eubacterium* | *-* |
|  |  |  | *Eubacterium MGBC000141* |
|  |  | *-* | *-* |
|  | *Lachnospiraceae* | *Dorea* | *Dorea MGBC117094* |
|  |  |  | *Dorea MGBC129764* |
|  |  |  | *-* |
|  |  | *Hominisplanchenecus* | *Hominisplanchenecus murintestinalis* |
|  |  | *Schaedlerella* | *Schaedlerella sp 000364245* |
|  |  |  | *-* |
|  |  | *Unclassified genus* | *Lachnospiraceae bacterium UBA3404* |
|  |  |  | *Lachnospiraceae bacterium UBA3401* |
|  | *Oscillospiraceae* | *Flavonifractor* | *Flavonifractor MGBC117744* |
|  | *Tannerellaceae* | *Parabacteroides* | *Parabacteroides johnsonii* |

| **Supplementary Table 3. Indicative bacterial list from the LEfSe analysis for homozygous 6-month-old** **male mice.** Only bacteria with an LDA score>2 and p<0.05 are shown. n=5/genotype | | | |
| --- | --- | --- | --- |
| **Sex and Genotype** | **Family** | **Genus** | **Species** |
| **APOE4 Males** | Clostridiaceae | Hungatella | Hungatella MGBC163323 |
|  |  |  | - |
|  | Erysipelotrichaceae | Dubosiella | Dubosiella newyorkensis |
|  |  |  | *-* |
|  | Eubacteriaceae | Eubacterium | Eubacterium MGBC130483 |
|  |  |  | Eubacterium MGBC122214 |
|  | Lachnospiraceae | Acetatifactor | Acetatifactor MGBC163656 |
|  |  |  | Acetatifactor muris |
|  |  | Anaerotignum | Anaerotignun MGBC136833 |
|  |  |  | - |
|  |  | Dorea | Dorea MGBC111235 |
|  |  |  | Dorea sp 000403455 |
|  |  |  | Dorea MGBC116392 |
|  |  | Kineothrix | Kineothrix MGBC162921 |
|  | Oscillospiraceae | Acutalibacter | Acutalibacter MGBC000533 |
|  |  |  | - |
|  |  | Angelakisella | Angelakisella MGBC161672 |
|  |  | Oscillibacter | Oscillibacter sp 1-3 |
|  |  |  | Oscillibacter MGBC163303 |
| **APOE3 Males** | Bacteroidaceae | Bacteroides | Bacteroides sp D22 |
|  | Clostridiaceae | Hungatella | Eubacterium MGBC107002 |
|  |  |  | Hungatella sp UBA3402 |
|  | Lachnospiraceae | Acetatifactor | Acetatifactor MGBC129690 |
|  |  | Schaedlerella | Schaedlerella MGBC163752 |
|  |  |  | Schaedlerella MGBC166500 |
|  |  | Unclassified genus | Lachnospiraceae bacterium A4 |
|  |  |  | Lachnospiraceae bacterium MD335 |
|  | Oscillospiraceae | Acutalibacter | Acutalibacter muris |
|  |  | Angelakisella | Angelakisella MGBC131977 |
|  |  |  | - |
|  |  | Butyricicoccus | Butyricicoccus MGBC131967 |
|  |  |  | *-* |
|  |  | Flavonifractor | - |
|  |  | Lawsonibacter | Lawsonibacter MGBC165440 |
|  |  | Oscillibacter | Oscillibacter MGBC129725 |
|  |  |  | Oscillibacter MGBC131504 |
|  |  |  | Oscillibacter MGBC163329 |
|  |  |  | Oscillibacter MGBC120045 |
|  |  |  | Oscillibacter MGBC115181 |
|  |  | Pseudoflavonifractor | - |
|  |  | Ruminiclostridium | Ruminiclostridium MGBC129722 |
|  |  |  | - |
|  |  | - | - |
|  | Turicibacteraceae | Turicibacter | Turicibacter sp UBA1159 |
|  |  |  | *-* |
|  |  | *-* | - |
| **APOE2 Males**  **APOE2 Males** | Akkermansiaceae | Akkermansia | Muribaculum sp 002492595 |
|  |  |  | *-* |
|  |  | - | *-* |
|  | Bacteroidaceae | Bacteroides | Bacteroides xylanisolvens |
|  |  |  | Bacteroides sp AM23-12 |
|  | Clostridiaceae | Hungatella | Hungatella MGBC000080 |
|  |  |  | Hungatella MGBC164450 |
|  | Coprobacillaceae | Thomasclavelia | Thomas claveliacocleata |
|  |  |  | - |
|  |  | *-* | *-* |
|  | Eubacteriaceae | Eubacterium | Eubacterium MGBC000141 |
|  |  |  | *-* |
|  |  | - | - |
|  | Lachnospiraceae | Dorea | Dorea MGBC117094 |
|  |  |  | Dorea MGBC129764 |
|  |  |  | Dorea MGBC000089 |
|  |  |  | *-* |
|  |  | Hominisplanchenecus | Hominisplanchenecus murintestinalis |
|  |  |  | - |
|  |  | Kineothrix | Kineothrix MGBC130615 |
|  |  | Lachnospira | Lachnospira MGBC164289 |
|  |  |  | *-* |
|  |  | Schaedlerella | Schaedlerella MGBC000001 |
|  |  |  | Schaedlerella sp000364245 |
|  |  |  | *-* |
|  |  | Unclassified genus | Lachnospiraceae bacterium UBA3404 |
|  |  |  | Lachnospiraceae bacterium UBA3401 |
